# The Effect of Oral Nimodipine on Cerebral Metabolism and Hemodynamic Parameters in Patients Suffering Aneurysmal Subarachnoid Hemorrhage

**DOI:** 10.1097/ANA.0000000000000928

**Published:** 2023-07-27

**Authors:** Miriam Moser, Yannik Schwarz, Johannes Herta, Walter Plöchl, Andrea Reinprecht, Markus Zeitlinger, Jonas Brugger, Dariga Ramazanova, Karl Rössler, Arthur Hosmann

**Affiliations:** Departments of * Neurosurgery; ‡ Clinical Pharmacology; † Department of Anesthesia, General Intensive Care Medicine and Pain Management; § Center for Medical Data Science, Medical University of Vienna, Austria

**Keywords:** aneurysmal subarachnoid hemorrhage, cerebral metabolism, cerebral microdialysis, cerebral perfusion pressure, multimodality neuromonitoring, nimodipine

## Abstract

**Introduction:**

Nimodipine is routinely administered to aneurysmal subarachnoid hemorrhage patients to improve functional outcomes. Nimodipine can induce marked systemic hypotension, which might impair cerebral perfusion and brain metabolism.

**Methods:**

Twenty-seven aneurysmal subarachnoid hemorrhage patients having multimodality neuromonitoring and oral nimodipine treatment as standard of care were included in this retrospective study. Alterations in mean arterial blood pressure (MAP), cerebral perfusion pressure (CPP), brain tissue oxygen tension (pbtO_2_), and brain metabolism (cerebral microdialysis), were investigated up to 120 minutes after oral administration of nimodipine (60 mg or 30 mg), using mixed linear models.

**Results:**

Three thousand four hundred twenty-five oral nimodipine administrations were investigated (126±59 administrations/patient). After 60 mg of oral nimodipine, there was an immediate statistically significant (but clinically irrelevant) drop in MAP (relative change, 0.97; *P*<0.001) and CPP (relative change: 0.97; *P*<0.001) compared with baseline, which lasted for the whole 120 minutes observation period (*P*<0.001). Subsequently, pbtO_2_ significantly decreased 50 minutes after administration (*P*=0.04) for the rest of the observation period; the maximum decrease was −0.6 mmHg after 100 minutes (*P*<0.001). None of the investigated cerebral metabolites (glucose, lactate, pyruvate, lactate/pyruvate ratio, glutamate, glycerol) changed after 60 mg nimodipine. Compared with 60 mg nimodipine, 30 mg induced a lower reduction in MAP (relative change, 1.01; *P*=0.02) and CPP (relative change, 1.01; *P*=0.03) but had similar effects on pbtO_2_ and cerebral metabolism (*P*>0.05).

**Conclusions:**

Oral nimodipine reduced MAP, which translated into a reduction in cerebral perfusion and oxygenation. However, these changes are unlikely to be clinically relevant, as the absolute changes were minimal and did not impact cerebral metabolism.

Outcomes after aneurysmal subarachnoid hemorrhage (aSAH) have improved over the last decades.^1,2^ However, delayed cerebral ischemia remains the major cause of poor functional outcome and death in aSAH patients.^2^ Oral nimodipine is the only drug approved for use with proven beneficial effects on functional outcome in aSAH patients.^3–8^ Nimodipine, a calcium channel blocker with high affinity for L-type voltage-gated calcium channels, counteracts the influx of calcium into vascular smooth-muscle cells, thereby leading to vasodilation with high specificity for cerebral arteries.^9,10^ It was initially believed to counteract the contraction of cerebral arteries during cerebral vasospasm, but several studies have shown that nimodipine does not reduce the frequency of vasospasm.^8,11–16^ Nowadays, a neuroprotective action is presumed because nimodipine reduces intracellular calcium, which plays an essential role in cellular apoptosis.^17^ This is important because delayed cerebral ischemia involves effects other than arterial narrowing, including neuroinflammation, microthrombosis, and cortical spreading depression.^18^ However, it remains unclear whether nimodipine can cross the blood-brain barrier to produce its putative neuroprotective effects. In addition, the vasodilatory effects of nimodipine can induce systemic hypotension, thereby potentially impairing cerebral perfusion.^19–28^ We hypothesize that nimodipine-induced hypotension might have a detrimental impact on cerebral metabolism and thereby outweigh its beneficial effects.

Therefore, this study was performed to investigate the effect of oral nimodipine on hemodynamic parameters (systemic arterial blood pressure and cerebral perfusion pressure) and cerebral oxygenation and metabolism (cerebral lactate, pyruvate, glucose, glutamate, and glycerol measured by cerebral microdialysis). The aim of the study was to evaluate whether nimodipine results in clinically relevant hypotension with concomitant cerebral hypoperfusion and metabolic failure.

## METHODS

In this explorative retrospective study of a prospectively collected database, all aSAH patients receiving oral nimodipine and requiring cerebral microdialysis monitoring as standard of care on the neurosurgical intensive care unit of the Medical University of Vienna between 2015 and 2019 were included. The study protocol was approved by the Ethics Committee of the Medical University of Vienna (EK-Nr. 1522/2017, amendment approval date: 26.05.2021).

### Clinical Management

All patients were managed according to an institutional protocol. Aneurysms were treated by clipping or coiling within the first 3 days after aSAH. In poor-grade patients an external ventricular drain was inserted upon arrival for cerebrospinal fluid diversion and control of intracranial pressure (ICP). Long-term sedation was established in patients with a decreased level of consciousness (Glasgow coma scale <9) and global cerebral edema with effacement of basal cisterns and hemispheric sulci for maximum cerebral protection. Initial sedation with continuous infusions of propofol and remifentanil was changed to sufentanil (up to 0.25 µg/kg/min) and midazolam (up to 20 mg/h) after 3 to 5 days to avoid the propofol infusion syndrome. In cases of insufficient sedation, ketamine infusion (up to 200 mg/h) was added. No neurological wake-up tests were performed during sedation to maximize cerebral protection.

Mean arterial blood pressure (MAP) was measured at the atrial level for clinical decision-making and at the tragus for calculation of cerebral perfusion pressure (CPP). Intracranial pressure was maintained <20 mmHg and brain tissue oxygen tension (pbtO_2_) >20 mmHg. Cerebral perfusion pressure thresholds were individualized guided by multimodality monitoring, but CPP was never allowed to fall below 60 mmHg. To maintain MAP and CPP targets, continuous norepinephrine infusion (up to 0.25 µg/kg/min during treatment with nimodipine 60 mg) was used. If target thresholds were not met, or there was a rapid reduction in MAP or CPP, the norepinephrine infusion rate was increased or the dose of nimodipine reduced.

### Multimodality Monitoring

All patients underwent cerebral multimodality neuromonitoring which included measurement of CPP, ICP, pbtO_2_, and neurochemistry (cerebral microdialysis). The indication for multimodal neuromonitoring was poor-grade aSAH or secondary deterioration requiring long-term sedation for cerebral protection.

Microdialysis probes with a membrane length of 10 mm and molecular weight cutoff of 20,000 Dalton, (70 MD Bolt Catheter or 70 Brain MD Catheter, M Dialysis AB, Stockholm, Sweden) were inserted in the middle/anterior cerebral artery territory ipsilateral to the ruptured aneurysm side or on the side with maximum subarachnoid blood extension. The microdialysis probes were perfused with artificial cerebrospinal fluid (Perfusion Fluid CNS, M Dialysis AB) at a flow rate of 0.3 µl/min using a microinfusion pump (107 Microdialysis Pump, M Dialysis AB). Microvials containing the microdialysate were collected every 1 to 2 hours and analyzed immediately for glucose, lactate, pyruvate, glutamate, and glycerol using a bedside analyzer (ISCUSflex, M Dialysis AB). The lactate to pyruvate ratio (LPR) was calculated.

A multimodality probe measuring ICP, pbtO_2_ and brain temperature (NEUROVENT-PTO 2L catheter, Raumedic AG) was inserted alongside the microdialysis probe. Multimodality neuromonitoring parameters (ICP, pbtO_2_, microdialysis variables and systemic hemodynamics) were stored every minute using the ICU pilot software (M Dialysis AB).

### Nimodipine Administration

Nimodipine 60 mg was started within 72 hours of hospital admission and administered through a gastric tube every 4 hours; it was continued until day 21, if tolerated. Continuous norepinephrine infusion was used in all patients to maintain target blood pressure values and avoid hypotension. In cases of therapy resistant hypotension (continuous norepinephrine infusion >0.25 µg/kg/min with 60 mg nimodipine 4 hourly) or clinical signs of pulmonal shunting the dose of nimodipine was reduced to 30 mg every 2 hours.

### Statistical Analysis

Patient demographics were reported as mean and SD for continuous variables and as total number and percentage of the study population for discrete variables. Baseline reference values for ICP, pbtO_2_, CPP, mean, systolic and diastolic arterial blood pressures were defined as the respective values observed 15 minutes before nimodipine administration. The latest observation between 0 and 50 minutes after nimodipine administration served as the baseline values for microdialysis parameters because of the time delay of 23 minutes in obtaining results due to the dead space of the microdialysis catheter outlet tube and the hourly change of the microvials.

For ICP, pbtO_2_, CPP, and mean, systolic and diastolic arterial pressures, observations from 0 to 120 minutes after each nimodipine administration were averaged in 10-minute intervals. For the microdialysis variables, all observed measurements between 50 and 140 minutes after nimodipine administration were averaged.

If the domain of the reference range of a parameter was constantly above zero, analyses were performed using the logarithm of the baseline value and averages after administration, as they were approximately normally distributed. This was the case for every parameter except ICP and pbtO_2_.

To investigate the effect of nimodipine on the respective outcomes, differences between baseline and post-dose 10-minute interval averages on the logarithmic scale were considered as the dependent variable in a linear model. The respective intervals and an indicator variable for nimodipine dose exposure (30 mg or 60 mg) were defined as explanatory variables. Only one interval after nimodipine administration was defined for microdialysis parameters and all models included a random effect for the patient.

For ICP, pbtO_2_, CPP, and mean systolic and diastolic arterial pressures, additional nested random effects for the number of nimodipine administrations within a patient were added to correct for potential residual effects of previous nimodipine administrations. Non-logarithmic values were used for ICP and pbtO_2_; thus, estimates correspond to absolute changes. Interactions between dose and time point were defined instead of a main effect for the dose load as the absolute effect of the dose could not be expected to be constant at all time points. However, the estimated difference of 30 mg doses of nimodipine was presented as an average over all time points for the sake of clarity. In addition, the same calculations were performed for all observation periods with critical cerebral perfusion, defined as pbtO_2_ <20 mmHg and CPP <60 mmHg at baseline before nimodipine administration.

Estimates and 95% CI and *P* values were computed for the null-hypotheses that absolute or relative changes were different from 0 or 1, respectively, for both nimodipine doses (30 mg and 60 mg) and whether estimated changes were different between doses. A *P* value <0.05 was considered statistically significant. No correction for multiple testing was applied; therefore, reported *P* values are of descriptive character. Statistical analysis was done using R, version 3.6.1 or higher.

## RESULTS

Three hundred twenty aSAH patients were treated at our institution during the study period; 27 (8.4%) received multimodal neuromonitoring and oral nimodipine and were included in this study. Baseline characteristics and outcomes of the included patients are shown in Table 1. On average, multimodality monitoring, including cerebral microdialysis, was started 4±4 days after aSAH and lasted for 14±5 days.

**TABLE 1:** Patients Characteristics, Outcomes and Multimodality Neuromonitoring

Patients’ characteristics	
Patients	27
Age	51±12
Sex, n (%)	
Female	21 (77.8)
Male	6 (22.2)
Hunt and Hess, n (%)	
Grade 2	3 (11.1)
Grade 3	5 (18.5)
Grade 4	10 (37.0)
Grade 5	9 (33.3)
Subarachnoid hemorrhage	
Ruptured aneurysm localization, n (%)	
Anterior communicating artery	9 (33.3)
Basilar artery	1 (3.7)
Middle cerebral artery	5 (18.5)
Posterior cerebral artery	1 (3.7)
Posterior communicating artery	5 (18.5)
Pericallosal artery	1 (3.7)
Posterior inferior cerebellar artery	2 (7.4)
Superior cerebellar artery	1 (3.7)
Multiple aneurysms with unclear rupture side	2 (7.4)
Surgical intervention, n (%)	
Clipping, n (%)	8 (29.6)
Coiling, n (%)	19 (70.4)
Surgical treatment after the bleeding event (d)	1±1.4
Start of multimodality monitoring after bleeding event (d)	4±4
Mean duration multimodality monitoring (d)	14±5
Side of probe, n (%)	
Left	13 (48.1)
Right	14 (51.9)
Infarction, n (%)	
No	10 (37.0)
Yes	17 (63.0)
Outcome	
Outcome after 3 mo GOS, n (%)*	
Grade 1	1 (3.8)
Grade 2	4 (15.4)
Grade 3	12 (46.2)
Grade 4	2 (7.7)
Grade 5	7 (26.9)
Mean multimodality monitoring parameters 0-120 minutes after nimodipine administration	
Mean arterial blood pressure [mmHg]	103.8±15.5
Systolic arterial blood pressure [mmHg]	155.9±25.4
Diastolic arterial blood pressure [mmHg]	74.8±11.7
ICP [mmHg]	7.7±4.9
CPP [mmHg]	85.9±16.7
pbtO2 [mmHg]	26.1±11.8
Glucose [mmol/l]	1.2± 0.6
Lactate [mmol/l]	4.3±1.6
Pyruvate (µmol/l)	118.3±39.2
Glutamate (µmol/l)	12.9±24.2
Glycerol (µmol/l)	88.0±128.1
Lactate/pyruvate ratio	37.6±9.5

* One patient was lost of follow-up.

CPP indicates cerebral perfusion pressure; GOS, Glasgow outcome scale; ICP, intracranial pressure; pbtO_2,_ brain tissue oxygen tension.

Hemodynamic and metabolic effects were investigated during 3425 nimodipine administrations. The average number of nimodipine administrations per patient was 126±59. In 61.3% of administrations, nimodipine 60 mg was used (77±47 administrations/patient), whereas nimodipine 30 mg was given in 38.7% of administrations (49±51 administrations/patient). The nimodipine 60 mg 4 hourly dosing regimen was not changed during the monitoring period in 2 (7%) patients, whereas there were multiple changes in nimodipine dosing in 17 (63%) patients. In 8 of these 17 patients (30%), the nimodipine dose was reduced from 60 to 30 mg at the end of the observation period. In addition to a dose reduction, nimodipine administration had to be paused for at least 1 day in 3 patients (11% of the 27 patients included in the study) due to therapy resistant hypotension or pulmonal shunting.

### Impact of Nimodipine on Multimodality Neuromonitoring

**FIGURE 1:**
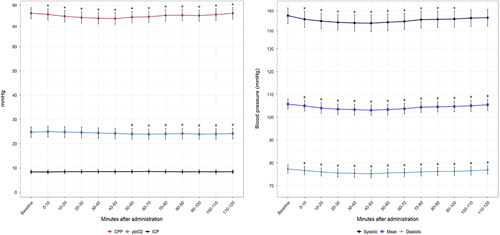
Changes in systemic hemodynamic, intracranial pressure, cerebral perfusion pressure, and brain tissue oxygen tension after nimodipine administration. Data are shown as average and standard error of the mean of patients’ median values in each interval. * statistically significant difference compared with baseline values according to the linear model. CPP indicates cerebral perfusion pressure; ICP, intracranial pressure; pbtO_2_, brain tissue oxygen tension.

Mean multimodality neuromonitoring parameters are shown in Table 1. There was a statistically significant decrease in MAP (relative change, 0.99; 95% CI, 0.984-0.996; *P*=0.001) and diastolic arterial pressure (relative change, 0.99; 95% CI, 0.985-0.997; *P*=0.002) immediately after administration of nimodipine 60 mg; this persisted for the entire 120 min observation period (Fig. 1 and Table 2). There was also an immediate and statistically significant reduction in systolic arterial pressure after nimodipine administration, but this had normalized after 100 minutes. The average decrease in MAP (relative change, 1.01; 95% CI, 1.001-1.016; *P*=0.02) and diastolic arterial pressure (relative change, 1.01; 95% CI, 1.002-10.16; *P*=0.01), but not in systolic arterial pressure (relative change, 1.01; 95% CI, 0.999-1.014; *P*=0.12), was significantly lower after administration of nimodipine 30 mg compared with nimodipine 60 mg (Table 2). Mean norepinephrine dose during nimodipine therapy was 0.084±0.049 µg/kg/min, and the maximum dose was 0.358±0.197 µg/kg/min.

**TABLE 2:** Absolute and Relative Statistically Estimated Changes of Hemodynamic and Multimodality Neuromonitoring Parameters Over Time After Administration of 60 mg and 30 mg of Nimodipine

Time	Mean blood pressure *P* *	Systolic blood pressure *P* *	Diastolic blood pressure *P* *	ICP (mmHg) *P* †	CPP *P* *	pbtO_2_ (mmHg) *P* †
0-10 min	0.99 [0.984, 0.996], 0.001	0.99 [0.986, 0.998], 0.01	0.99 [0.985, 0.997], 0.002	0.0 [−0.1, 0.1], 0.91	0.99 [0.98, 0.99],<0.001	0.2 [−0.2, 0.5], 0.31
10-20 min	0.99 [0.976, 0.988],<0.001	0.98 [0.978, 0.990],<0.001	0.98 [0.976, 0.987],<0.001	0.1 [−0.1, 0.2], 0.28	0.98 [0.97, 0.98],<0.001	0.2 [−0.2, 0.5], 0.33
20-30 min	0.97 [0.968, 0.980],<0.001	0.98 [0.972, 0.984],<0.001	0.97 [0.969, 0.980],<0.001	0.1 [−0.1, 0.2], 0.31	0.97 [0.96, 0.97],<0.001	0.1 [−0.3, 0.4], 0.75
30-40 min	0.97 [0.967, 0.979],<0.001	0.98 [0.972, 0.984],<0.001	0.97 [0.967, 0.978],<0.001	0.0 [−0.1, 0.1], 0.69	0.97 [0.96, 0.97],<0.001	−0.2 [−0.5, 0.2], 0.30
40-50 min	0.97 [0.969, 0.980],<0.001	0.98 [0.974, 0.986],<0.001	0.97 [0.968, 0.980],<0.001	0.0 [−0.1, 0.2], 0.73	0.97 [0.96, 0.98],<0.001	−0.3 [−0.6, 0.1], 0.11
50-60 min	0.98 [0.971, 0.983],<0.001	0.98 [0.976, 0.988],<0.001	0.98 [0.972, 0.983],<0.001	0.0 [−0.1, 0.2], 0.53	0.97 [0.97, 0.98],<0.001	−0.3 [−0.7, −0.0], 0.04
60-70 min	0.98 [0.973, 0.985],<0.001	0.98 [0.978, 0.990],<0.001	0.98 [0.974, 0.985],<0.001	0.0 [−0.1, 0.2], 0.94	0.98 [0.97, 0.98],<0.001	−0.4 [−0.8, −0.1], 0.01
70-80 min	0.98 [0.977, 0.989],<0.001	0.99 [0.982, 0.994],<0.001	0.98 [0.978, 0.989],<0.001	0.0 [−0.1, 0.2], 0.65	0.98 [0.97, 0.99],<0.001	−0.4 [−0.8, −0.1], 0.01
80-90 min	0.99 [0.980, 0.992],<0.001	0.99 [0.984, 0.996], 0.002	0.99 [0.980, 0.992],<0.001	0.1 [−0.1, 0.2], 0.45	0.98 [0.98, 0.99],<0.001	−0.5 [−0.8, −0.1], 0.004
90-100 min	0.99 [0.982, 0.994],<0.001	0.99 [0.985, 0.997], 0.01	0.99 [0.982, 0.993],<0.001	0.1 [−0.1, 0.2], 0.28	0.99 [0.98, 0.99],<0.001	−0.6 [−0.9, −0.3],<0.001
100-110 min	0.99 [0.984, 0.996], 0.002	0.99 [0.988, 1.000], 0.07	0.99 [0.984, 0.996],<0.001	0.1 [−0.1, 0.2], 0.39	0.99 [0.98, 1.00], 0.003	−0.6 [−1.0, −0.3],<0.001
110-120 min	0.99 [0.987, 0.999], 0.02	1.00 [0.990, 1.002], 0.21	0.99 [0.987, 0.999], 0.02	0.1 [−0.1, 0.2], 0.35	0.99 [0.98, 1.00], 0.02	−0.5 [−0.8, −0.2], 0.004
Mean difference after administration of 30 mg compared with 60 mg nimodpine	1.01 [1.001, 1.016], 0.02	1.01 [0.999, 1.014], 0.12	1.01 [1.002, 1.016], 0.01	0.03 [−0.15, 0.21], 0.75	1.01 [1.00, 1.02], 0.03	0.23 [−0.16, 0.62], 0.24

Data presented as estimated relative or estimated absolute differences from baseline [95% CI].

* Indicates an estimated relative differences to baseline.

† Indicates an estimated absolute difference to the baseline.

CPP indicates cerebral perfusion pressure; ICP, intracranial pressure; pbtO_2_, brain tissue oxygen tension.

There were no changes in ICP during the 120 minutes observation period after administration of both 60 mg and 30 mg nimodipine (Fig. 1 and Table 2). After nimodipine 60 mg, there was an immediate decrease in CPP below baseline values; this reduction in CPP reached its maximum 20 minutes after nimodipine administration (relative change, 0.97; 95% CI, 0.96- 0.97; *P*<0.001) and persisted for the whole 120 minutes observation period (Fig. 1 and Table 2). After administration of nimodipine 30 mg, the reduction in CPP was, on average, lower compared with that after nimodipine 60 mg (estimated at 1.01 times the value after 60 mg; 95% CI, 1.00-1.02: *P*=0.03); however, statistically significant 10 to 70 minutes after nimodipine.

**FIGURE 2:**
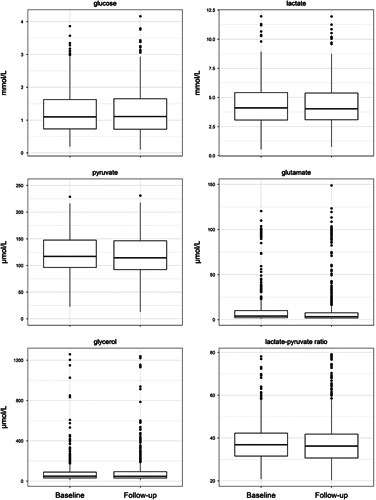
Cerebral metabolites before and after nimodipine administration. Boxplots showing the median and the upper and lower quartile for cerebral metabolite before and after administration of nimodipine 60 mg. The whiskers represent 1.5 times the interquartile range from the respective quartile. Outliers outside this range are presented as black dots. If no outliers were present, the whiskers end at the minimum of the observations.

There was a statistically significant decrease in pbtO_2_ 50 minutes after nimodipine 60 mg, which persisted throughout the 120 minutes observation period (Fig. 1 and Table 2); the maximum decrease in pbtO_2_ occurred 100 minutes after nimodipine administration (−0.7 mmHg; 95% CI, −0.9 to −0.3 mmHg). There were no significant changes in pbtO_2_ after administration of 30 mg nimodipine in comparison to 60 mg (increase of 0.2; 95% CI, −0.2 to 0.6; *P*=0.24) (Table 2). There were no significant changes in cerebral metabolites (cerebral glucose, pyruvate, lactate, LPR, glutamate and glycerol) after both 60 mg and 30 mg of nimodipine (Fig. 2 and Table 3).

**TABLE 3:** Estimated Relative Change of Cerebral Metabolites After Administration of Nimodipine 60 mg and Relative Change of Cerebral Metabolites After 30 mg Nimodipine Compared With 60 mg

	Relative change after 60 mg nimodipine	*P*	Relative change after 30 mg compared with 60 mg nimodipine	*P*
Glucose (mmol/l)	1.01 [0.99; 1.03]	0.380	1.01 [1.00, 1.05]	0.498
Lactate (mmol/l)	1.00 [0.99; 1.02]	0.777	1.01 [1.00, 1.03]	0.659
Pyruvate (µmol/l)	1.00 [0.99; 1.02]	0.958	1.01 [1.00, 1.03]	0.524
Glutamate (µmol/l)	1.00 [0.95; 1.05]	0.896	1.00 [1.00, 1.07]	0.971
Glycerol (µmol/l)	1.01 [0.99; 1.03]	0.348	1.00 [1.00, 1.02]	0.435
Lactate/pyruvate Ratio	1.01 [1.00; 1.02]	0.225	1.00 [1.00, 1.02]	0.752

Data presented as relative change [95% CIs].

In patients with critical cerebral perfusion (pbtO_2_ <20 mmHg and CPP <60 mmHg), mean pbtO_2_ was 8.9±6.7 mmHg and CPP 55 ±4.1 mmHg at baseline before nimodipine administration. Interestingly in these patients pbtO_2_ had increased significantly by 70 to 80 min after nimodipine administration (increase of 1.81 mmHg; 95% CI, 0.651-2.097 mmHg; *P*=0.002) and this increase was maintained up to 120 minutes post-administration (*P*<0.001); the maximum increase in pbtO_2_ occurred at 80 to 90 minutes after nimodipine administration (increase of 2.17 mmHg; 95% CI, 1.01-3.33 mmHg; *P*=0.001). Cerebral perfusion pressure was also increased 60 to 120 minutes after nimodipine administration with a maximum increase compared with baseline after 80 minutes (relative increase, 1.106; 95% CI. 1.03-1.18; *P*=0.004) (Supplemental Digital Content 1, http://links.lww.com/CJP/A1000: Table showing changes in pbtO_2_ and CPP in patients with baseline critical cerebral perfusion). There were no significant changes in microdialysis parameters in patients with critical cerebral perfusion before nimodipine administration.

## DISCUSSION

In this retrospective study using a prospectively collected database, multimodality neuromonitoring parameters including cerebral metabolic variables were investigated in 27 aSAH patients after oral nimodipine administration. Nimodipine induced a significant decrease in arterial blood pressure and CPP (with stable ICP) and a subsequent reduction in pbtO_2_. However, these alterations were of minimal clinical relevance as the parameters remained within physiological limits when the nimodipine-related hypotension was counteracted with vasopressors. Consequently, cerebral metabolism was not compromised after oral administration of nimodipine.

As a potent vasodilator, nimodipine can induce marked hypotension.^19,21–27^ In aSAH patients with impaired cerebral autoregulation, it is crucial to maintain an adequate MAP to maintain adequate brain perfusion and avoid cerebral hypoxia and cerebral metabolic crisis.^22^ Only poor-grade aSAH patients requiring long-term sedation were included in this study, a cohort which is especially prone to severely impaired autoregulation and risk of hypotension-induced cerebral hypoperfusion. Choi et al^19^ also reported a statistically significant decrease in MAP and CPP after oral nimodipine administration despite vasopressor treatment. Similar to our results, these changes were of no clinical significance; the absolute reduction in MAP was in single digits and the reduction in CPP was only 1.2 mmHg lower than baseline, and did not result in any clinically relevant effects on pbtO_2_ (decrease of 1.03 mmHg) or cerebral blood flow (decrease of 0.39 mL/100 g/min).^19^

Interestingly, in a prospective study including 11 patients, Stiefel et al^28^ found that MAP and CPP remained unchanged after oral nimodipine administration, though there was a significant reduction in pbtO_2_. Again, though changes were statistically significant, the pbtO_2_ remained within physiological limits. Nevertheless, these findings suggest that oral nimodipine can not only induce a reduction in systemic blood pressure but might also impair the cerebral microcirculation resulting in the reduction in pbtO_2_; however, unlike in our study, cerebral microdialysis measurements were not available to evaluate actual cerebral metabolic state in the study by Stiefel et al.^28^

Cerebral metabolic variables, including LPR and cerebral glucose, were measured in addition to MAP, CPP, and pbtO_2_ in the study by Choi et al.^19^ Glucose is an important energy substrate and cerebral glucose concentration is an indirect marker of cerebral perfusion.^29^ In contrast to our results, Choi et al^19^ found a significant increase in cerebral glucose concentration after nimodipine administration, suggesting an improvement in cerebral energy supply; however, similar to our results, the LPR did not change in that study. For the accurate evaluation of cerebral metabolism, knowledge of absolute cerebral lactate and pyruvate levels, as well as the LPR, are crucial. Pyruvate is the metabolic end product of glycolysis and essential for mitochondrial energy formation. In the event of ischemia, pyruvate is transformed to lactate resulting in an increased LPR. However, the LPR can also be elevated because of mitochondrial dysfunction, which is frequently observed after aSAH.^30^ In contrast to cerebral ischemia, sufficient substrate (i.e., pyruvate and oxygen), is available during mitochondrial dysfunction.^31,32^ In the present cohort, cerebral pyruvate and pbtO_2_ remained within their physiological ranges, but cerebral lactate levels increased in most patients. This resulted in an overall increase in the LPR, suggesting mitochondrial dysfunction rather than ischemia. None of these metabolic parameters changed after nimodipine administration, suggesting that nimodipine has neither a positive nor negative effect on cerebral metabolic state. Therefore, the metabolic derangement observed in our study was most likely related to the underlying severity of the aSAH rather than to nimodipine administration.

To further investigate cerebral metabolism after nimodipine administration, cerebral glutamate and glycerol levels were also included in our analysis. Cerebral glutamate is a very sensitive and early marker of energy depletion and thereby an indirect marker of cerebral ischemia.^32–35^ We found no changes in cerebral glutamate levels after oral nimodipine administration, suggesting that there was no cerebral compromise. Cerebral glycerol is an indicator of cell membrane breakdown and therefore a marker of manifest ischemia.^32^ Although cerebral glycerol was elevated in some patients in our study, it did not change after nimodipine administration. Therefore, cerebral metabolism seemed not to be compromised by nimodipine administration.

Despite identifying statistically significant changes, the hemodynamic and metabolic effects of oral nimodipine were not clinically significant in our study. However, when interpreting these results, the specific clinical management must be considered. The association between low blood pressure and metabolic crisis emphasizes the need for strict vasopressor management to counteract any nimodipine-induced hypotension and minimize the risk of cerebral compromise.^22^ All patients in this study received continuous norepinephrine infusion to maintain adequate MAP. Although nimodipine-induced reductions in systemic blood pressure could not be completely prevented, vasopressor use ensured that the reductions in blood pressure were at clinically irrelevant levels.

The safe use of norepinephrine to facilitate full dosage of nimodipine was reported by Pala et al^23^ in a retrospective analysis of 397 poor-grade (World Federation of Neurological Surgeons grade IV or V) aSAH patients in which not only nimodipine but also the vasopressor dose, were independent predictors of good 6-month functional outcomes.^23^ Thus, even high dose vasopressor use is reasonable to allow the crucial goal of administration of a maximum dose of nimodipine in aSAH patients; this is particularly important in poor-grade patients as included in our cohort. According to our institutional protocol, excessive doses of norepinephrine (>0.25 µg/kg/min) were avoided in our study to minimize severe side effects.^36–38^ Therefore, in cases of marked hypotension despite maximal vasopressor therapy, the dose of nimodipine was reduced from 60 mg every 4 hours to 30 mg every 2 hours. Such dose reduction was necessary at least once in 93% of the patients in this study and nimodipine was paused for at least 1 day in 11% of the patients. In the literature, a dose reduction of oral nimodipine has been reported in only 28% to 49% of patients compared with 93% in the present cohort, and paused in 29% compared with 11% in this study.^24,25^ Similar to our clinical management, nimodipine dose was reduced to maintain adequate MAP despite maximal vasopressor therapy in one study.^25^ In that study, a nimodipine dose reduction because of intractable hypotension was more likely to occur in patients with more severe aSAH.^25^ Marked hypotension occurs more often in poor grade than in good grade aSAH patients;^27^ therefore, the inclusion of poor grade and deeply sedated aSAH patients in our study cohort might explain the higher frequency of nimodipine dose reductions. Nevertheless, our clinical management allowed continuation of nimodipine treatment with a low incidence of complete therapy withdrawal. Discontinuation of nimodipine is an independent predictor for delayed cerebral ischemia and should be avoided.^26^ A nimodipine dose reduction to 30 mg every 2 hours hardly changed hemodynamic parameters and cerebral metabolism compared with 60 mg in our cohort, and thus seems to be safe. However, the clinical efficacy of a reduced nimodipine dosage regime remains unclear.

According to our data, oral nimodipine does not induce acute cerebral ischemia in poor-grade aSAH patients; although the hemodynamic changes observed were statistically significant they clinically irrelevant because of the minimal hemodynamic effects. Therefore, use of 60 mg nimodipine seems to be safe. However, cerebral metabolism was still deranged because of the underlying aSAH, as we observed metabolic parameters above physiological values. In future prospective trials, it would be interesting to evaluate the effects of nimodipine in relation to impaired cerebrovascular autoregulation assessed by the pressure reactivity index.^39^ Moreover, it is unknown whether different doses or routes of nimodipine administration might improve cerebral metabolism. Although higher dose nimodipine might improve microcirculation due to its vasodilative effects, it might also have more pronounced effects on systemic hemodynamics. Although this can be counteracted by vasopressors, it remains unclear what maximal dose of norepinephrine should be tolerated to allow continuation of nimodipine therapy. Intravenous administration of nimodipine results in a higher bioavailability in plasma than oral administration;^40,41^ however, a beneficial impact of nimodipine on functional outcomes has only been proven for oral nimodipine, challenging a dose-dependent effect.^7^ Increasing nimodipine target site concentration within brain parenchyma might improve its assumed neuroprotective effects.^17^ Therefore, further studies are needed to prospectively analyze whether different doses and routes of nimodipine administration (oral, intravenous, or intra-arterial) achieve sufficient target site concentrations within the brain and improve microperfusion and cerebral metabolism.

This study has several limitations. First, although we used a prospectively collected database, parameters analyzed in the study were collected retrospectively with the known limitations of such an approach; data are dependent on reported details, some data are missing, and nimodipine administrations were not timed with the microdialysis measurements. Second, though the adequacy of cerebral perfusion was estimated with CPP, pbtO_2_, and microdialysis measurements, actual cerebral blood flow was not measured. Third, microdialysis and pbtO_2_ measurements are focal and limited to the brain region surrounding the probe; therefore, the probes were placed into the watershed of the anterior/middle cerebral artery territories of the more affected brain hemisphere to monitor both vascular territories of the vulnerable side. However, nimodipine-induced systemic hypotension affects the brain globally, and some brain regions remote from the monitoring probes might be more affected than the regions surrounding the probes. Fourth, the drift of the intraparenchymal ICP monitors over time cannot be excluded; however, the reported drift of such technology is minimal and mostly recorded at prolonged use over 20 days.^42^ Fifth, the sample size was limited to 27 patients. Although our department is a tertiary referral center for cerebrovascular diseases, the number of patients requiring both invasive multimodality neuromonitoring and oral nimodipine administration was limited. Sixth, this was an explorative study so no statistical power analysis was performed. Finally, this was a single center study and the institutional treatment protocols must be considered when interpreting our data.

In conclusion, oral nimodipine induced a statistically significant but clinically irrelevant reduction in MAP, CPP, and pbtO_2_. Cerebral metabolism was not impaired as CPP and pbtO_2_ remained within their physiological ranges when blood pressure decreases were counteracted with vasopressors. Therefore, the current protocol for the use of nimodipine in poor-grade aSAH patients seems to be safe in terms of its impact on cerebral perfusion and metabolism. Future studies designed to investigate the safety and efficacy of different nimodipine doses and routes of administration to improve both cerebral metabolism and hemodynamics are required.

## Supplementary Material

SUPPLEMENTARY MATERIAL
