# Supplementary material for: The Effect of Oral Nimodipine on Cerebral Metabolism and Hemodynamic Parameters in Patients Suffering Aneurysmal Subarachnoid Hemorrhage
Source: J Neurosurg Anesthesiol. 2023 Jul 27;36(4):317–25. doi: 10.1097/ANA.0000000000000928 (PMC11377055; doi:10.1097/ANA.0000000000000928)
Supplement: SUPPLEMENTARY MATERIAL [file ana-36-317-s001.docx]

**Supplementary Table 1**

**Estimated changes of pbtO_2_ and CPP over time after administration of 60 mg or 30 mg of nimodipine during episodes of critical perfusion (pbtO_2_ < 20 mmHg and CPP < 60 mmHg).**

* indicates an estimated relative differences and ^#^ an estimated absolute difference to the baseline

| **Time** | **pbtO_2_ (mmHg) [95% CI], p-value** ^#^ | **CPP [95% CI], p-value** ^*^ |
| --- | --- | --- |
| 0-10 minutes | 0.5 [-0.7, 1.7], 0.4 | 1.06 [0.99, 1.13], 0.11 |
| 10-20 minutes | 0.6 [-0.5, 1.8], 0.3 | 1.07 [1.00, 1.14], 0.07 |
| 20-30 minutes | 0.8 [-0.4, 2.0], 0.2 | 1.06 [0.99, 1.14], 0.08 |
| 30-40 minutes | 1.0 [-0.2, 2.1], 0.1 | 1.06 [0.99, 1,13], 0.12 |
| 40-50 minutes | 1.0 [-0.2, 2.1], 0.1 | 1.05 [0.98, 1.12], 0.17 |
| 50-60 minutes | 0.9 [-0.3, 2.0], 0.1 | 1.06 [0.99, 1.13], 0.11 |
| 60-70 minutes | 0.9 [-0.2, 2.1], 0.1 | 1.08 [1.01, 1.16], **0.02** |
| 70-80 minutes | 1.8 [0.7, 3.0], **0.002** | 1.10 [1.03, 1.18], **0.01** |
| 80-90 minutes | 2.2 [1.0, 3.3], **<0.001** | 1.11 [1.03, 1.18], **0.004** |
| 90-100 minutes | 2.0 [0.8, 3.1], **<0.001** | 1.10 [1.03, 1.18], **0.01** |
| 100-110 minutes | 2.0 [0.9, 3.2], **<0.001** | 1.10 [1.03, 1.18], **0.01** |
| 110-120 minutes | 2.0 [0.8, 3.1], **<0.001** | 1.10 [1.02, 1.17], **0.01** |
| Estimated mean difference 30mg dose | -0.2 [-1.8, 1.3], 0.8 | 1.01 [0.92, 1.10], 0.85 |
